# MWCNTs-GNPs Reinforced TPU Composites with Thermal and Electrical Conductivity: Low-Temperature Controlled DIW Forming

**DOI:** 10.3390/mi14040815

**Published:** 2023-04-04

**Authors:** Chenqi Duan, Fei Long, Xiaolu Shi, Yuting Wang, Jiajing Dong, Songtao Ying, Yesheng Li, Yuchuan Cheng, Jianjun Guo, Gaojie Xu, Aihua Sun

**Affiliations:** 1 Ganzhou Key Laboratory of Advanced Metals and Functional Materials, School of Materials Science and Engineering, Jiangxi University of Science and Technology (JXUST), 86 Hongqi Road, Ganzhou 341000, China; 2 Key Laboratory of Additive Manufacturing Materials of Zhejiang Province, Ningbo Institute of Materials Technology and Engineering, Chinese Academy of Sciences, Ningbo 315201, China; 3 Department of Mechanical, Materials and Manufacturing Engineering, University of Nottingham, Ningbo 315100, China; 4 Ningbo New Material Testing and Evaluation Center Co., Ltd., Ningbo 315201, China

**Keywords:** direct ink writing, low temperature control, bifunctional, thermoplastic polymer

## Abstract

As an effective technique for fabricating conductive and thermally conductive polymer composites, a multi-filler system incorporates different types and sizes of multiple fillers to form interconnected networks with improved electrical, thermal, and processing properties. In this study, DIW forming of bifunctional composites was achieved by controlling the temperature of the printing platform. The study was based on enhancing the thermal and electrical transport properties of hybrid ternary polymer nanocomposites with multi-walled carbon nanotubes (MWCNTs) and graphene nanoplates (GNPs). With thermoplastic polyurethane (TPU) used as the matrix, the addition of MWCNTs, GNPs and both mixtures further improved the thermal conductivity of the elastomers. By adjusting the weight fraction of the functional fillers (MWCNTs and GNPs), the thermal and electrical properties were gradually explored. Here, the thermal conductivity of the polymer composites increased nearly sevenfold (from 0.36 W·m^−1^·k^−1^ to 2.87 W·m^−1^·k^−1^) and the electrical conductivity increased up to 5.49 × 10^−2^ S·m^−1^. It is expected to be used in the field of electronic packaging and environmental thermal dissipation, especially for modern electronic industrial equipment.

## 1. Introduction

With excellent comprehensive performance that integrates the characteristics of high strength, light weight, corrosion resistance, ease of processing and low processing costs [1,2,3], polymer materials are widely applied in the fields of consumer electronics, automotives, aerospace, etc. For example, thermoplastic polyurethane film with good mechanical properties can be used as a stretchable member of a new type of shock isolator [4]. The continuous updating of various devices puts forwards more demands on the functions of materials. During the operation of electronic equipment, the heat per unit volume generated continues to increase, and this accumulated heat seriously affects the reliability of device operation and greatly reduces the service life of electronic devices [5,6]. Therefore, the related thermal management systems need to satisfy higher requirements. Particularly, the trend of automotive lightweighting is driving the demand for conductive polymer products in the automotive industry. Conductive polymer products are being used to replace metal parts in some components, which can solve the problems associated with electrostatic discharge damage, high weight of metal parts, and high cost. In addition, these polymers are resistant to electromagnetic interference (EMI) and radio frequency interference (RFI) [7,8,9]. The antistatic packaging segment is a key demand field for conductive polymer products as packaging issues are the leading cause of product damage, and approximately one-third of damage to electronic and electrical products is related to electrostatic discharge [10,11]. As a result, the growing demand for antistatic packaging is driving the demand for conductive polymer products.

Due to the poor electrical insulation and thermal properties, most polymers have limited application in engineering, and the previous studies merely concentrated on enhancing the single function of polymer composites. In general, the thermal conductivity of polymers is about 0.1–0.5 W·m^−1^·K^−1^ [12,13,14]. There are two primary methods used to improve the thermal conductivity of polymers. One is to obtain intrinsically thermally conductive polymers by improving the material crystallinity and the orientation of molecular chains, and another is to synthesize the filled thermally conductive polymers by mixing highly thermally conductive fillers with the polymer matrix. Intrinsic-type polymer preparation is time-consuming, expensive, and challenging to scale up. Researchers have enhanced the thermal conductivity of polymers by adding inorganic materials with high thermal conductivity (e.g., carbonaceous materials, boron nitride, silicon nitride, and metals) [15,16,17,18,19,20,21]. Guo et al. [22] constructed a three-dimensional hybrid filler system via the surface modification of MWCNTs as well as GNPs, which reduced the interfacial thermal resistance between the filler and the matrix. The modified results greatly improved the interfacial compatibility between the filler and the substrate, and prepared highly thermally conductive poly(vinylidene fluoride) (PVDF) composites that were improved by 711% compared with pure PVDF. The most common method used to improve the electrical conductivity of polymers is to prepare filled composite conductive materials by adding conductive fillers. Sun et al. [23] has fabricated Mxene@PDMS nanocomposites with low percolation thresholds through electrostatic assembly and compression molding. These materials exhibited excellent electrical conductivity and EMI shielding performances throughout the x-band (>54 dB).

Integrated bifunctional lightweight materials with good thermal and electrical conductivity are urgently needed to enhance safety and to extend the lifetime of electronic devices. Conventional processes such as injection molding, melt extrusion or thermal induction face huge challenges in efficiently manufacturing products with complex geometries and customized designs for applications in aerospace and biomedical industries [24,25]. Additive manufacturing (AM), a new type of material forming, is well positioned to overcome these problems. Especially, direct ink writing (DIW) has become one of the most commonly used AM methods to create complex and periodic 3D structures using a wide range of materials [26,27]. In contrast to other AM processes, DIW is not limited by material class as long as the precursor ink shows specific rheological properties [28]. DIW has been used to fabricate thermoset polymers of any shape with the desired properties and functions [29,30,31]. Guo et al. [32] has studied the solvent-based 3D printing process of PLA solutions and investigated the process-dependent viscosity of PLA solutions using capillary flow analysis. In addition, a range of processing parameters for fabricating different microstructures has been proposed. Their research focused on how to use this method to shape material without performing further research into functional polymer composites.

In this work, the solvent ink of thermoplastic polymer TPU is selected as the printing matrix, and the conductive and thermally conductive properties of the matrix are enhanced by adding nano-functional fillers such as GNPs and MWCNTs. Meanwhile, the DIW formation of TPU is controlled by controlling the temperature of the printing platform. Due to the inclusion of two different morphological nanofillers, the synthesized TPU has excellent properties of electrical and thermal conductivity. The interaction between these two nanofillers with different shapes favors the formation of an interconnected network inside the matrix. This method can be considered as an economical and simple approach with which to obtain printed components with both electrically and thermally conductive functionalities, as well as a new technique for fabricating complex structures from thermoplastic polymer solutions. It is expected to be used in the field of electronic packaging and environmental thermal dissipation, especially for use in modern electronic industrial equipment.

## 2. Experimental Section

### 2.1. Materials

Thermoplastic polyurethane (TPU, Elastollan 1185A, BASF Co., Ltd.) was chosen as the flexible polymer matrix. N, N-dimethylformamide was used as a solvent (DMF, Sinopharm Chemical Reagent Co., Ltd., China). The highly conductive MWCNTs powder used in this work was provided by Carbene Technology Co., Ltd. (Shenzhen, China); the highly thermally conductive GNPs powder used in this work was provided by Xiamen Knano graphene Technology Co., Ltd.

### 2.2. Polymer Solution Preparation

Firstly, MWCNTs and GNPs were dispersed in DMF under mechanical stirring to form a uniformly dispersed suspension. Next, the TPU particles were completely dissolved in the DMF solvent by use of mechanical stirring. Then, the suspension was gradually added to the TPU solution and mechanical stirring was continued for more than 6 h. Next, the solution was poured into a Petri dish and placed in a fume hood to evaporate the solvent, and then the obtained viscous ink was stirred in a planetary gravity stirrer (2000 rpm, 1.0 KPa) for 4 min to remove the generated air bubbles, and finally the inks for DIW printing were obtained. The ink used for printing needs to have the characteristic of shear thinning. Shear thinning is the non-Newtonian behavior of a fluid in which the viscosity decreases as the shear rate increases. As a result, even at lower extrusion pressures, it aids the ink to flow through the nozzle, ensuring extrudability.

### 2.3. 3D Printing

**Figure 1:**
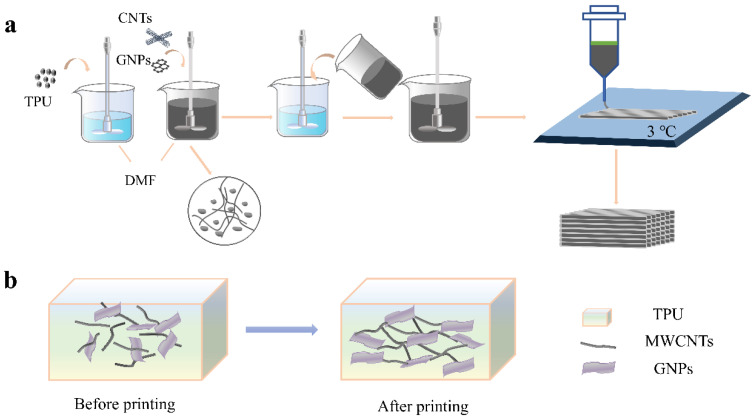
(**a**) Ink formulation and DIW forming process. (**b**) Schematic diagram of the filler arrangement mechanism inside the polymer matrix.

All samples were printed using the same multi-material Bio 3D printer (REGENOVO 3D Bio-Architect^®^ Sparrow; Hangzhou Regenovo Biotechnology Co., Ltd., China). A 610 μm nozzle was used to connect the syringe filled with configured ink with the extrusion pressure of 0.25 MPa and the printing speed was controlled to 4–7 mm·s^−1^ by optimizing the printing parameters. The entire procedure for the DIW printing of electrically and thermally conductive materials has been depicted in Figure 1a. Firstly, a homogenous solution is created by thoroughly dissolving the TPU particles in the DMF solution. To achieve greater dispersion of the functional fillers in the polymer matrix, the fillers are disseminated in a DMF solution to form a homogeneously dispersed suspension. The fabrication of DIW printing inks involves three main steps to remove air bubbles from the ink, namely mechanical agitation, solvent evaporation, and vacuum agitation. By designing the structure and determining the printing path, pre-set samples can be obtained.

### 2.4. Characterization

Rheological measurements were performed using a rotational rheometer (HAAKE R6000, Thermo Electron GmbH, Karlsruhe, Germany) with a 15 mm diameter parallel-plate geometry. A field emission scanning electron microscopy device (FESEM, S-4800, Hitachi, Japan; Verios G4 UC, Thermo Scientific, USA) was used to observe the morphology of the samples. Large samples were freeze-fractured in liquid nitrogen and sputtered with platinum. The thermal diffusion coefficient of the samples was measured with a laser thermal conductivity meter (LFA467, NETZSCH, Germany). The drainage method was applied to measure the density of the composites. An LCR digital bridge (TH2878, Tong-Hui, Changzhou, China) was used to measure the electrical conductivity of the samples, and conductive silver paste was applied to the ends of the samples.

## 3. Result and Discussion

### 3.1. Rheological Properties of Bifunctional Composite Inks

**Figure 2:**
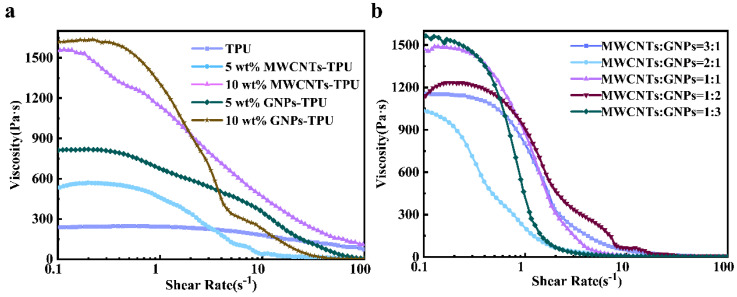
Rheological properties of different components and different filling proportions of ink. (**a**) Viscosity curves of TPU inks filled with pure TPU and single component fillers. (**b**) Viscosity curves of composite inks at room temperature using a total of 10 wt% fillers with different proportions of MWCNTs and GNPs fillers (MWCNTs:GNPs = (3:1, 2:1, 1:1, 1:2, 1:3)).

To achieve the desired structures for stable 3D printing, DIW composite inks with specific rheological properties need to be configured. The initial viscosity of a TPU solution varies greatly with different concentrations, which influences the printing parameters [32]. Here, a TPU solution of 700 mg·mL^−1^ was chosen as the initial concentration for printing, and the selected nanoscale functional fillers, GNPs and MWCNTs, possess excellent electrical and thermal conductivity properties. Incorporating these functional fillers into the polymer matrix enables us to improve the electrical and thermal conductivity of the polymers significantly. As shown in Figure 2a, the increase in filler weight fraction will enlarge the initial viscosity of composite inks gradually. The viscosity of TPU solutions with different proportions of functional filler exhibits a typical shear thinning behavior over a range of shear rates, the ink being transformed from a high-viscosity state during the shear process. At a low shear rate, the polymer internal molecular chains are irregularly entangled with each other, which causes certain types of damage. However, the destruction rate is not high, being roughly equal to the generation rate, and thus it shows a constant viscosity. When the shear rate is close to 0, the flow characteristics are similar to those of Newtonian fluid, and viscosity tends to be constant. When the shear rate exceeds a certain critical shear rate, shear thinning behavior is exhibited. This region is a typical flow region found in polymer material processing, and is called a non-Newtonian flow region or shear thinning region. The shear thinning behavior exhibited in this region ensures smoother extrusion of the ink from inside the nozzle. In addition to enhancing the functionality of the polymer, the fillers can also give the polymer some support. It has been verified by experiments that the shape of the sample printed by the solution with lower filler content cannot be maintained well, and also that the shrinkage is too serious after drying. On the other hand, the solution with higher filler content is not as smooth as the solution with a low concentration when printing. After the sample was completely dry, the sample flexibility was greatly reduced. As shown in Figure S1, the printing display of 15 wt% GNPs-TPU ink is shown. The ink is printed with the same diameter needle, the same printing speed is 4 mm·s^−1^, and the extrusion pressure is 0.5MPa. The surface of the printed sample is much roughed than that of the 10 wt% GNPs-TPU. Therefore, a TPU solution with a filler weight fraction of 10 wt% was selected for further optimization. When adding one type of filler (MWCNTs or GNPs), the higher filler amounts can lead to the composite having lower flexibility and electrical insulation properties. Mixtures of fillers with different morphologies and sizes can play a positive role, constructing more thermally and electrically conductive pathways within the polymer matrix. Therefore, the sum of MWCNT content and GNP content equal to 10 wt% was selected to configure the ink, MWCNTs:GNPs = (3:1, 2:1, 1:1, 1:2, 1:3), respectively.

As shown in Figure 2b, the viscosities of the five inks with different ratios show obvious shear thinning behavior in a certain range of shear rate, which can ensure smooth extrusion from the head of the needle. However, there is no specific rule for the initial viscosity of two-component filler printing inks, and their initial viscosities do not show an obvious change pattern with the increase in a component percentage. The experimental results demonstrate that the ink with MWCNTs:GNPs = 1:3 has the highest initial viscosity, while the ink with MWCNTs:GNPs = 2:1 has the lowest initial viscosity. The main reason for this result is the interaction of two fillers with different shapes. Specially, the morphology of nanocomposites is a complex issue as agglomeration, exfoliation, interactions, etc., might exist between particles, which may lead to irregular variations in the initial viscosity. In addition, the applied solvent is DMF with a certain volatility, which partially affects the viscosity determination when performing the rheological testing of printing inks on a rotational rheometer. However, during the printing process, the upper part of the syringe used to hold the printing ink is closed, and so the solvent does not evaporate in the syringe during the printing process.

**Figure 3:**
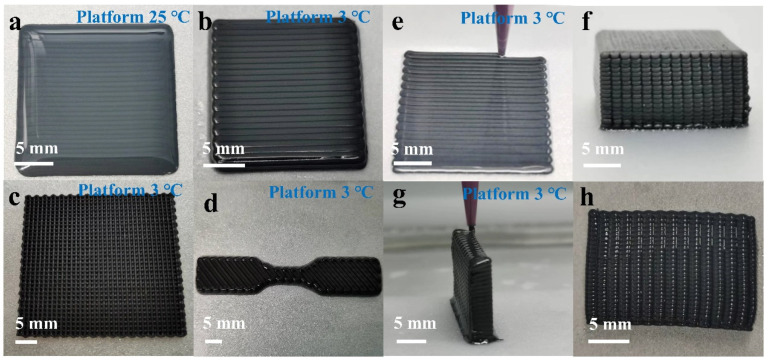
Printing 20 mm × 20 mm × 1 mm samples with temperatures at (**a**) 25 °C and (**b**) 3 °C; (**c**) 40 mm × 40 mm grid samples and (**d**) dumbbell shape samples formed at 3 °C; (**e**) 20 mm × 20 mm × 10 mm samples in the printing process and (**f**) after completed printing; (**g**) the printing process and (**h**) the printed sample of 4 mm × 20 mm × 12 mm. The above applied inks are all 10 wt% GNPs-TPU.

The designed inks satisfy the shear thinning rheological requirements. This may be established by examining the rheological parameters involved. Additionally, the printing effect of the ink has been verified through assessment of the printed solid sample. As shown in Figure 3a–d, the samples are shown under different printing environments, respectively. The sample in Figure 3a was printed at a room temperature of 25 °C (the temperature of the printhead and the printing platform were kept at the same temperature as the ambient temperature, and printing parameters were set with a conical needle diameter of 610 μm, a filling pitch of 700 μm, and a pneumatic pressure of 0.25 MPa). Instead of maintaining their initial shapes after printing for a while, the samples collapsed in all directions, while the boundary between each filament gradually disappeared until they finally merged together. To maintain the original shape of the sample, we were attempted to control by adjusting the temperature of the platform. As shown in Figure 3b, when the platform temperature was 3 °C, the sample was able to maintain its original printed shape for the period that ran from the completion of printing to the completion of curing. Since the TPU used in this experiment is a thermoplastic that is solid at room temperature, the TPU was dissolved in an organic solvent to form a polymer solution. The polymer solution product does not have the property of viscoelastic inversion that the material extruded from the needle itself does not provide support As shown in Figure 3a, the sample did not undergo curing after a period of time after printing. This occurred primarily because the DMF used in this experiment is an organic solvent. It belongs to the continuous phase of the composite ink. After the sample had been printed, the solvent was still in a flowing state inside the printed sample. Therefore, the experiment was required to make use of external conditions to maintain the shape of the sample. The process of controlling the temperature of the printing platform to maintain the shape of the printed sample was implemented. First of all, the temperature of the printing platform was controlled at 0 °C, and it was found that the sample was able to maintain a good shape. However, the temperature difference of the printing environment was too large, which would have led to the condensation of water vapor on the sample. As such, the temperature was controlled at 3 °C, and the sample could also maintain a good shape. To verify the reliability of the method, the same ink was used to print the samples of different shapes with the same printing parameters and different filling methods (Figure 3c,d), and they all were found to maintain good printed shapes and structures after curing. The suitable low temperatures were more conducive to maintaining the shape of the printed sample. We analyzed the reasons as to why the shape can be maintained under the control of low temperature. One reason is that the solubility of the polymer matrix in the solvent is reduced at low temperature, leading to partial solvent precipitation. This process is irreversible when the temperature is maintained. It may also be because the fluidity of the solvent is reduced at low temperature, meaning that the sample retains its shape when printing.

With high aspect ratios, both 1D-structured MWCNTs and 2D-structured GNPs exhibit relatively excellent performances in polymer composites. In terms of nanoscale high thermal conductivity fillers such as MWCNTs and GNPs, it is necessary on the one hand to solve the problem of interfacial thermal resistance caused by size effect, and on the other hand to improve the disorder distribution by aggregation. Effective dispersion can be regarded as the key to achieving the best thermal conductivity for polymer composites. This quality can be improved by the use of filler synergy, external field induction, and prefabricated 3D thermal conductivity networks [33]. The synergy between two materials of various sizes is depicted in Figure 1b, in which the MWCNTs mainly act as bridges to connect the GNPs to form an interoperable network for enhancing matrix performance. During printing, GNPs tend to have a more parallel alignment in the composite, resulting in higher thermal conductivity along the printing direction than that along the vertical one. As shown in Figure 3e–h, the printed and finished samples were measured for electrical and thermal conductivity. Furthermore, polytetrafluoroethylene (PTFE) molds were used to cast various ink components in different proportions, and the ink was poured into the molds for evaporating solvent before casting the samples into shape. The molded sample, as well as the printed and fully cured sample, were then placed in a vacuum drying oven at 45 °C for over 24 h to remove any residual solvent. The final sample was obtained for testing using various molding methods.

### 3.2. Morphological Characterization

**Figure 4:**
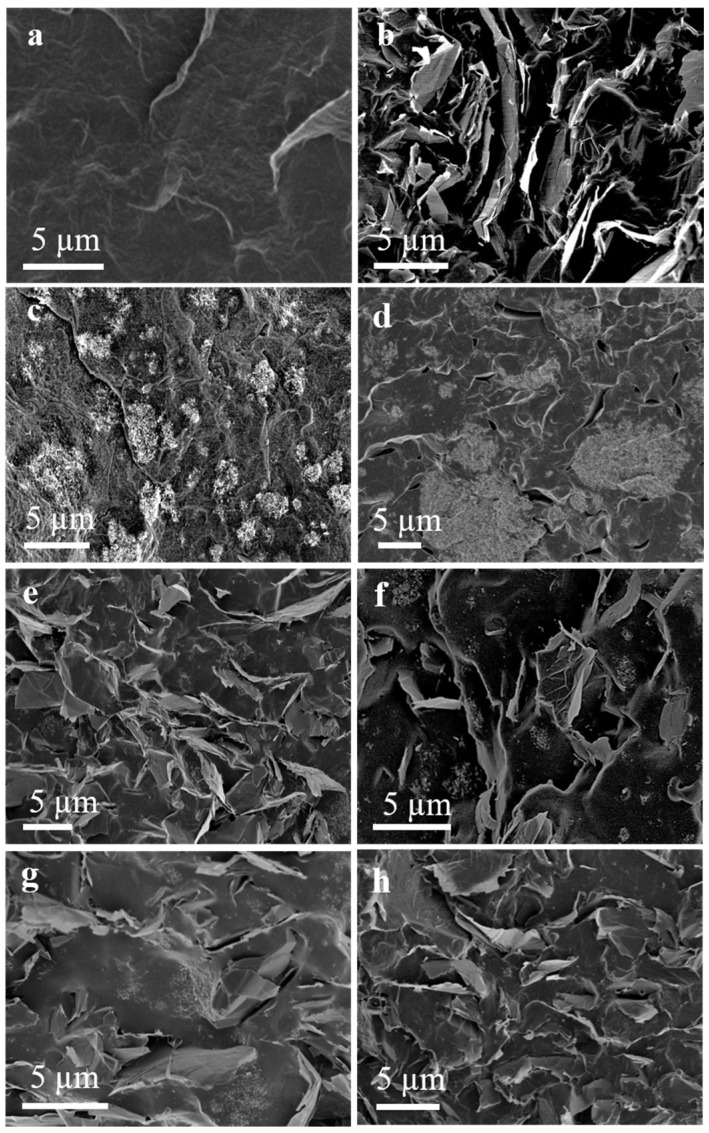
SEM cross-sections of TPU composites. DIW forming sample: (**a**) Pure TPU; (**b**) 10 wt% GNPs-TPU; (**c**) 10 wt% MWCNTs-TPU; (**d**) mold forming of 10wt% (MWCNTs:GNPS = 1:2)-TPU; DIW forming sample: (**e**) 10 wt% (MWCNTs:GNPs = 3:1)-TPU; (**f**) 10 wt% (MWCNTs:GNPs = 2:1)-TPU; (**g**) 10 wt% (MWCNTs:GNPs = 1:1)-TPU; (**h**) 10 wt% (CNTs:GNPs = 1:2)-TPU.

The morphologies of the MWCNTs and GNPs powder embedded in the TPU matrix were investigated using scanning electron microscopy (SEM), as shown in Supplementary Figure S2. The fracture surfaces of pure TPU (Figure 4a) were used as the standards to compare with the TPU nanocomposites, adding different proportions of functional materials. In the preset direction of printing nozzle action, the uniform distribution and alignment of GNPs in the 10 wt% GNPs-TPU composites revealed how GNPs interacted to form a local seepage network, while the MWCNTs of the 10 wt% MWCNTs-TPU nanocomposite were not well dispersed within the TPU matrix with a serious agglomeration. As materials with high aspect ratios, MWCNTs endowed the composites with excellent properties but brought problems of poor dispersion and agglomeration. With the increase in GNPs (Figure 4e–h), the fillers built a better interconnection network in which the properties within the matrix were better. This indicated that GNPs and MWCNTs can prevent each other from aggregating, and can thus improve the dispersion in their respective TPU matrix. Long and curved MWCNTs such as arms and bridging GNPs may inhibit their aggregation, leading to the formation of strongly interconnected hybrid nanofiller networks in the polymer matrices. In the high-magnification micrograph (Figure S3), it can be observed that MWCNTs act as interconnections which form a permeable network between GNP planes. With respect to the MWCNTs/GNPs-TPU nanocomposites, the MWCNTs play roles as conductive flexible pathways spanning between the larger high-surface area GNPs. These two different shapes of fillers interacted to form a mixed percolation network, which was expected to enhance the electrical and thermal conductivity of the samples. The SEM images of the molded samples (Figure 4d) showed the agglomeration of a large number of MWCNTs. These could not form and align with the internal graphene in the DIW printed samples, but were only able to do so through the thin crack morphology in their matrix.

### 3.3. Thermal Conductivity and Electrical Conductivity of Printed Samples

**Figure 5:**
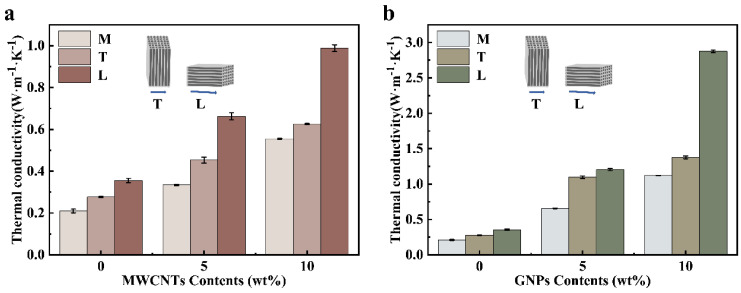
Single component thermal conductivity of (**a**) pure TPU, 5 wt% MWCNTs-TPU, 10 wt% MWCNTs-TPU; (**b**) pure TPU, 5 wt% GNPs-TPU, 10 wt% GNPs-TPU.

The laser flash method is used to evaluate the thermal conductivity of various kinds of samples. The method involves a test sample test being designed as a square (10 × 10 mm or 6 × 6 mm), with a thickness in 1–2 mm. In this study, M, T (transverse) and L (longitudinal) (illustration in Figure S4) represent the thermal conductivity of the mold, both perpendicular to the printing direction (perpendicular to the XY plane direction) and along the printing direction (perpendicular to the YZ plane direction), respectively. As shown in Figure 5a, with the increase in MWCNTs, the thermal conductivity of the polymer composites tends to increase, and the thermal conductivity of each component varies with the molding methods and the orientations of the sample. For molded samples, an increase in filler from 0 to 10 wt% resulted in an increase of 166% in thermal conductivity from 0.21 W·m^−1^·K^−1^ to 0.56 W·m^−1^·K^−1^. Regarding the printed sample, under the same operation conditions, the thermal conductivity in the T direction increased from 0.28 W·m^−1^·K^−1^ to 0.63 W·m^−1^·K^−1^, an increase of 125%. The thermal conductivity in the L direction increased from 0.36 W·m^−1^·K^−1^ to 0.98 W·m^−1^·K^−1^, an increase of 166%. As shown in Figure 5b, the thermal conductivity of the polymer composites was on an increasing trend with growing GNP growing content, and the thermal conductivity of each component varied with the molding method and sample orientation. We found that 10 wt% GNPs-TPU composites showed the most significant change in thermal conductivity. For the molded samples, after a filler up to 10 wt% had been added, the thermal conductivity increased from 0.21 W·m^−1^·K^−1^ to 1.12 W·m^−1^·K^−1^, an increase of 433%. For the print-formed samples, the thermal conductivity in the T direction and L direction increased by 393% (28 W·m^−1^·K^−1^ to 1.38 W·m^−1^·K^−1^) and 697% (0.36 W·m^−1^·K^−1^ to 2.87 W·m^−1^·K^−1^), respectively. In the system with a single-component filler, GNPs had better thermal conductivity, and the thermal conductivity in the L direction was roughly twice that in the T direction. The above findings are consistent with the SEM findings displayed in Figure 4. Although the MWCNTs had an excellent thermal conductivity in the MWCNTs-TPU composite, the high interfacial thermal resistance between the MWCNTs and TPU as well as MWCNT agglomeration prevent the formation of a large number of continuous pathways in the polymer matrix. Furthermore, the contact thermal resistance of MWCNTs at the inter-tube junction was an important factor affecting the thermal conductivity of MWCNTs-filled polymer composites.

**Figure 6:**
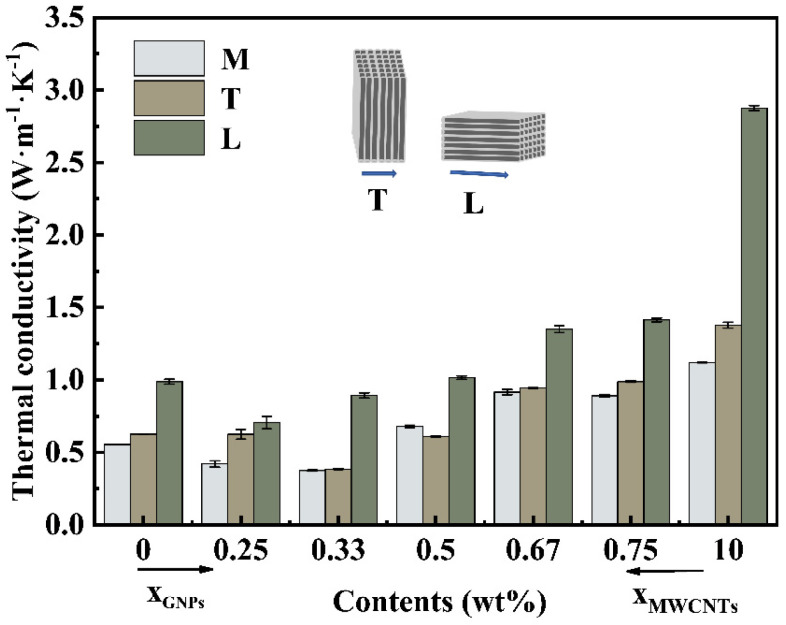
Thermal conductivity of two-component filled samples with different ratios and different forming methods.

Interfacial defects between the filler and the polymer matrix can lead to phonon scattering to such an extent that the thermal conductivity with the composite becomes not ideal. Hybrid filler combinations were introduced to combine fillers of different shapes, sizes, and compositions in order to form layered thermal conductivity paths and overcome the problem of severe filler agglomeration in the matrix of single-filler systems [34]. The effect of the two-component fillers on the thermal conductivity of the polymer composites was investigated and the results are shown in Figure 6. The *x* axis represented the mass fraction of GNPs and MWCNTs in the polymer composite, and the total content of GNPs and MWCNTs was 10 wt%. MWCNTs-TPU, MWCNTs:GNPs = (3:1, 2:1, 1:1, 1:2, 1:3), and GNPs-TPU are shown in order from left to right. The experimental data indicate that the thermal conductivity of the two-component filler polymer composites gradually increased with the ratio of GNPs, and there was a certain positive effect between the two fillers with different shapes. However, the thermal conductivity of the dual-filler system was not as high as that of GNPs-TPU, and the influence of the filler in the polymer matrix was hindered by the weak interfacial bonds between the two phases. Additionally, there was higher thermal resistance between MWCNTs and the polymer matrix, and inherently higher contact thermal resistance between MWCNTs and GNPs followed as a consequence. In 1D MWCNTs compared with 2D GNPs, the van der Waals forces between MWCNTs led to easy entanglement or agglomeration into bundles, which caused problems in the distribution of carbon nanotubes in solution. The addition of GNPs alleviated this problem. In detail, this is because they played a role as a surfactant to prevent the formation of agglomerates.

**Figure 7:**
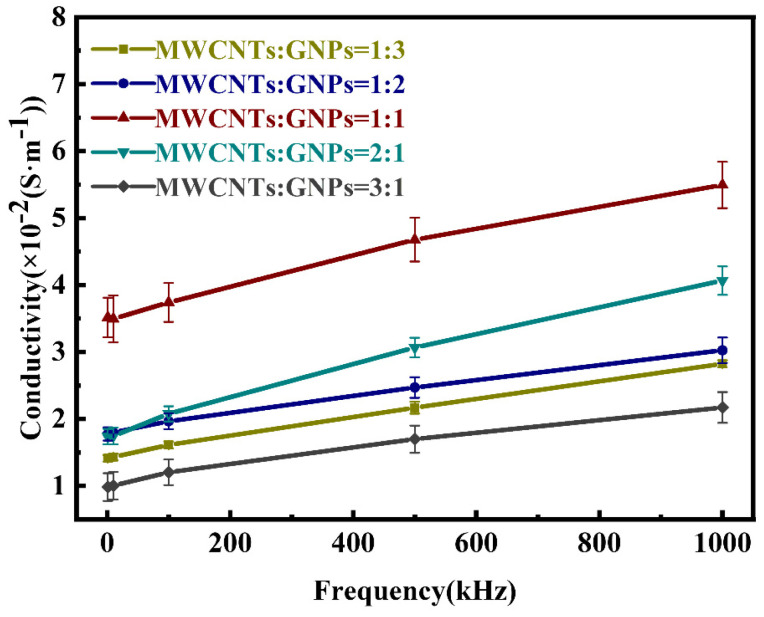
Conductivity of a print-shaped sample filled with two components of different ratios.

When measuring the conductivity of the sample, the implementation direction is the one in which printing occurs. We first applied conductive silver paste to both ends of the sample which was to be tested in order to remove the static charge which had accumulated on the contact area. The 10 wt% two-component-filled conductive composites were tested at different frequencies in the range of 1–1000 kHz and at frequencies of 1 kHz, 10 kHz, 100 kHz, 500 kHz, and 1000 kHz. The conductivity test results at different frequencies for each ratio are shown in Figure 7. The conductivity of MWCNTs:GNPs in the equal weight fraction was higher with 3.51 × 10^−2^ S·m^−1^ at 1 kHz. As the frequency increased, the conductivity of this ratio also increased until the frequency had rised to 1000 kHz, with a conductivity of 5.49 × 10^−2^ S·m^−1^. This was an increase of nearly 56%. The electrical conductivity (10^−2^~10^0^ S·m^−1^) of polymer-based conductive composites for circuit components and cable semi-conductive layers has thus been satisfied, and we expect it to be applied in practice. Additionally, the electrical conductivity did not show frequency dependence (resistive behavior) at lower frequencies in the measure range, and the conductivity change, followed by increasing frequency, showed frequency dependence (capacitive behavior). Therefore, it can be judged that the conductivity of the composite is due to a combination of physical contact among fillers (dominant at low frequencies) and adjacent non-contact fillers through electron tunneling and electron leap mechanisms (dominant at high frequencies). The total mass fraction of fillers was the same, but there was a great variation in conductivity. However, it did not vary with the proportion of particular fillers. The conductivity increased with the growth of GNPs content, and the conductivity peaked when MWCNTs: GNPs had equal weight fractions. The conductivity of MWCNTs/GNPs-TPU composites was influenced by a wide variety of factors, such as the processing method, the degree of exfoliation and dispersion of the filler, the orientation of the filler, and the aspect ratio of the filler, etc. Compared with the melt compounding method, the solution compounding method is more conducive to the exfoliation of GNPs sheets, which helps to improve the overall conductivity. As far as the aspect ratio of the filler is concerned, the rod-shaped fillers are more effective in improving the conductivity than the flake-shaped material for the same aspect ratio. However, compared with GNPs, the rod-shaped MWCNTs were more easily entangled in the polymer matrix, as shown in Figure 4 and the SEM images (Figure S3). The MWCNTs always aggregated easily, which was more obvious in the mold-formed samples than that in the DIW print-formed samples. In addition, comparing the GNPs in Figure S2 with those in Figure 4, it can be observed that the shear mixing force breaks some of the flakes into smaller pieces or distorts their shapes, which subsequently increases the exfoliation of the GNPs and also reduces the effective aspect ratio of some GNPs. Therefore, the difference in conductivity is not caused by a single factor, but by a combination of a series of factors.

## 4. Conclusions

In this research, thermoplastic polymer composite sample parts can be prepared by the DIW method through the manipulation of ink concentration and printing platform temperature. The discovery of such TPU materials provides a new concept for printing thermoplastic polymers with complex structures of nanofillers with high thermal properties and electrical conductivity. This can effectively enhance the performance of composite materials, especially when combined with the DIW printing fabrication method. Along the printing direction (perpendicular to the YZ plane), the thermal conductivity of the molded sample increases up to 2.87 W·m^−1^·K^−1^, which is nearly sevenfold higher than before, and the electrical conductivity can reach 5.49 × 10^−2^ S·m^−1^. A comparison of this work with published work of the same type is presented in Table S1. This facile DIW printing approach can be used to fabricate thermoplastic polymer composite devices with complex structures used in electronic packaging and external environment thermal dissipation which are capable of both electrical and thermal conduction.

## Data Availability

Data supporting the findings of this study are available from the corresponding author upon reasonable request.

## Supplementary Materials

The following supporting information can be downloaded at: https://www.mdpi.com/article/10.3390/mi14040815/s1, Figure S1: (a) and (b) Printed display of 15 wt% GNPs-TPU samples at 3 °C (the extrusion pressure of 0.5 MPa and the printing speed was controlled among 4 mm·s^−1^); Figure S2: SEM images of (a) MWCNTs powder and (b) GNPs powder; Figure S3: SEM cross-section images of (a) 10 wt% (MWCNTs:GNPs = 2:1)-TPU and (b) 10 wt% (MWCNTs:GNPs = 1:1)-TPU; Figure S4: (a) Slicing diagram of sample T. (b) slicing diagram of sample L; Table S1. Comparison between this work and the published related work.
