# Supplementary material for: MWCNTs-GNPs Reinforced TPU Composites with Thermal and Electrical Conductivity: Low-Temperature Controlled DIW Forming
Source: Micromachines (Basel). 2023 Apr 4;14(4):815. doi: 10.3390/mi14040815 (PMC10144802; doi:10.3390/mi14040815)
Supplement: Supplementary file 1 [file micromachines-14-00815-s001.zip › micromachines-2290098-supplementary.pdf]

Supplementary Materials

MWCNTs-GNPs Reinforced TPU Composites with Thermal and Electrical Conductivity: Low-temperature Controlled DIW Forming

Chenqi Duan [1, 2], Fei Long [2,3], Xiaolu Shi [4], Yuting Wang [2], Jiajing Dong [2], Songtao Ying [2], Yesheng Li[1], Yuchuan Cheng [2], Jianjun Guo [2], Gaojie Xu [2] and Aihua Sun [2]**

[1]Ganzhou Key Laboratory of Advanced Metals and Functional Materials, School of Materials Science and Engineering, Jiangxi University of Science and Technology (JXUST), 86 Hongqi Road, Ganzhou 341000, PR China.

[2]Key Laboratory of Additive Manufacturing Materials of Zhejiang Province, Ningbo Institute of Materials Technology and Engineering, Chinese Academy of Science, Ningbo 315201, PR China

E-mail: *nfyyliyesheng@163.com* *sunaihua@nimte.ac.cn*

[3]Department of Mechanical, Materials and Manufacturing Engineering, University of Nottingham, Ningbo 315100, P. R. China

[4]Ningbo New Material Testing and Evaluation Center Co. Ltd, Ningbo 315201, PR China

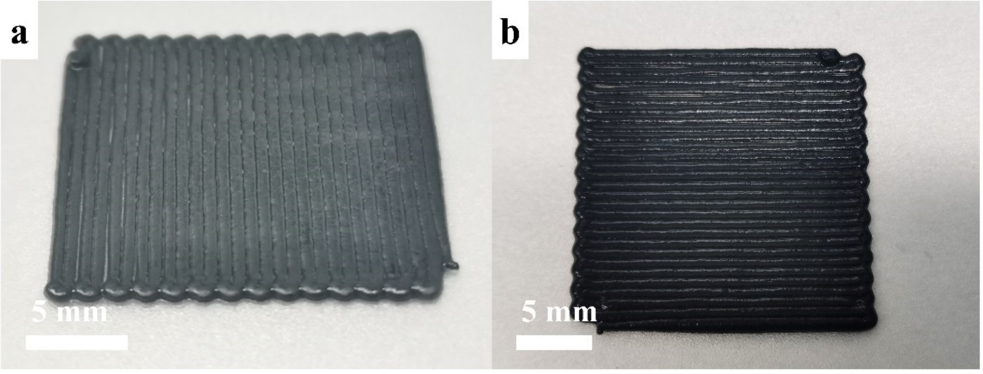

Figure S1. a) and b) Printed display of 15 wt% GNPs-TPU samples at 3 °C (the extrusion pressure of 0.5 MPa and the printing speed was controlled among 4 mm·s^{-1})

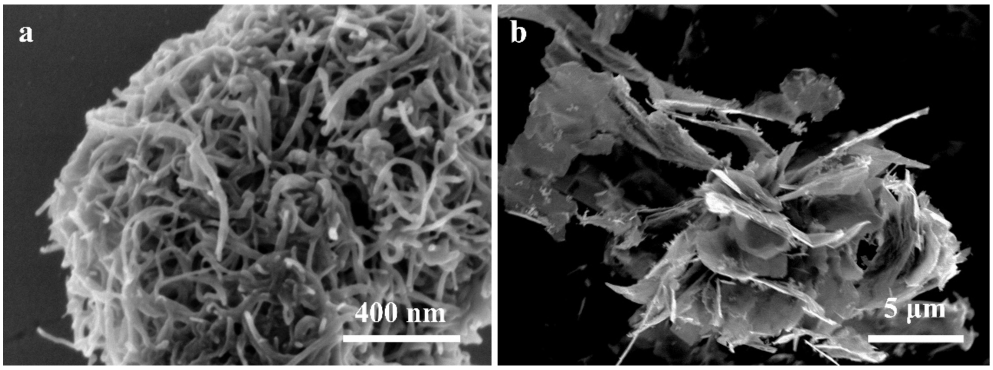

Figure S2. SEM images of a) MWCNTs powder and b) GNPs powder.

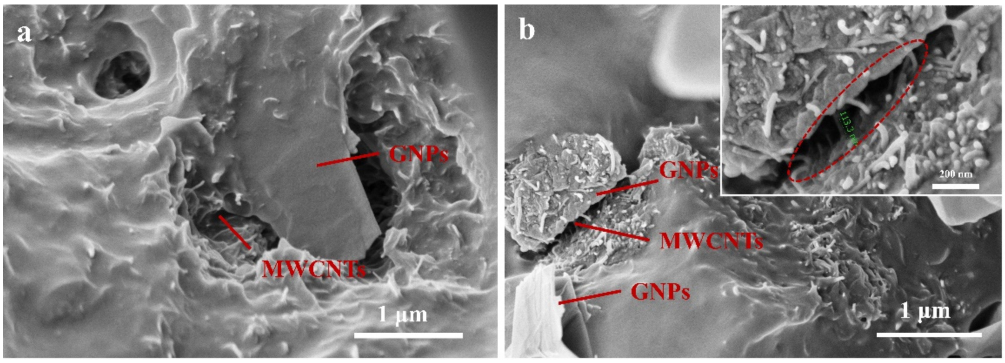

Figure S3. SEM cross-section images of a)10 wt% (MWCNTs: GNPs=2:1)-TPU and b) 10 wt% (MWCNTs: GNPs=1:1)-TPU.

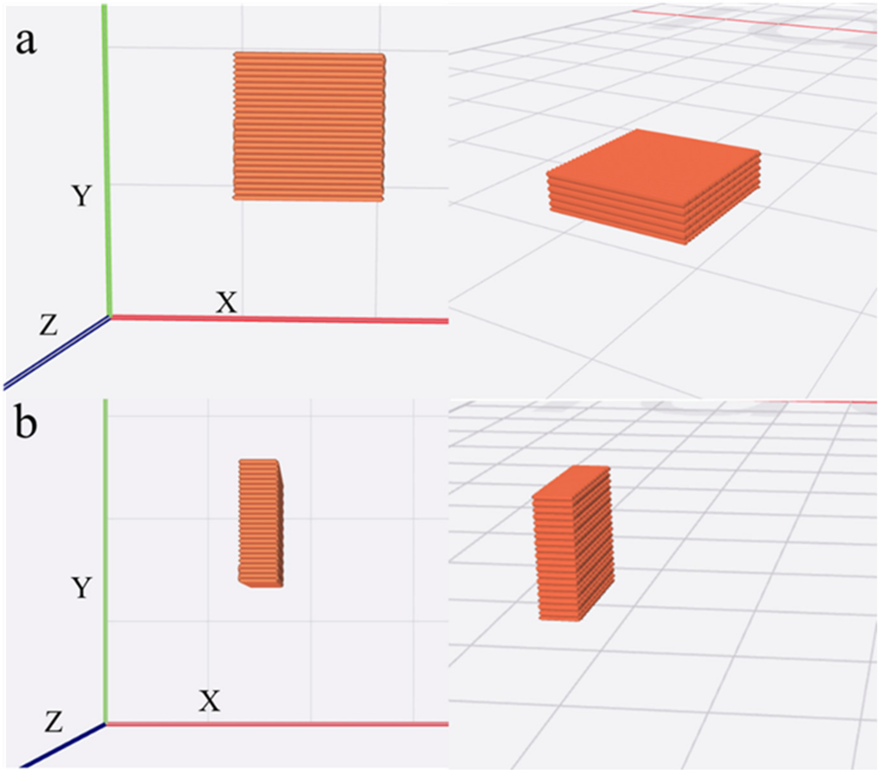

Figure S4. a) Slicing diagram of sample H. b) slicing diagram of sample V.

The test instrument requires the test sample test part area to be square (10 mm × 10 mm or 6 × 6 mm), and the sample thickness in 1-2mm. as shown in Figure S4, all samples are printed along the X-axis, and each layer is printed in the same way. The heat conduction direction to be tested for sample T (Figure S4a) is along the Z axis, and the heat conduction direction to be tested for sample L (Figure S4b) is along the X axis. Sample M is molded in a mold of 40 mm×40 mm×2 mm and cut to the required size during use.

Table S1. Comparison between this work and the published related work.

Polymer	Filler Content	Thermal Conductivity λ [$W\cdot M^{-1}\cdot k^{-1}$]	Electrical Conductivity σ [$S\cdot m^{-1}$]	Notes	Ref
TPU	10 wt% GNPs	2.87	/	DIW	This work
	10 wt% (MWCNTs: GNPs=1:1)	0.81	5.49×10^{-2}		
TPU	2 wt% (CNTs: GNPs=3:1)	/	1.92×10^{-3}	FDM	[1]
TPU	3 wt% CNTs	/	4.45×10^{-3}	FDM	[2]
TPU	45 wt% GNPs	12	/	FDM	[3]
PA6/POE-g-MAH/PS	50 wt% Graphite	5.5	/	FDM	[4]
TPU	5 wt% MWCNTs	0.55	/	FDM	[5]
PLA	1.2 wt% GNPs	/	0.1	FDM	[6]
PEEK	10 wt% CNTs	$0.4 < \lambda < 0.43$	/	FDM	[7]
TPU	1 wt% GNPs	0.41	/	FDM	[8]
PA12	5 wt% MWCNTs	/	$1 < \sigma < 10$	FDM	[9]
	12 wt% MWCNTs	0.365	4.54		
PLA	12 wt% GNPs	0.664	6.27	FDM	[10]
	12 wt% (MWCNTs: GNPs=1:1)	0.533	0.95		
PA12	15 wt% GNPs +1wt%CNTs	0.73	/	FDM	[11]

Table S1 summarizes and compares this work with published work. There are many types of research on 3D printing using GNPs and MWCNTs as functional fillers, and there are also many alternative printing methods. However, the research on thermoplastics as matrix basically adopts the way of FDM, and the researches on printing functional thermoplastic polymer composites with DIW are almost no. Therefore, we chose to contrast with FDM.

References

[1] D. Xiang, X. Zhang, Z. Han, Z. Zhang, Z. Zhou, E. Harkin-Jones, J. Zhang, X. Luo, P. Wang, C. Zhao, Y. Li, *Journal of Materials Science* **2020**, *55,*15769.

[2] D. Xiang, X. Zhang, Y. Li, E. Harkin-Jones, Y. Zheng, L. Wang, C. Zhao, P. Wang, *Composites Part B: Engineering* **2019**, *176*,107250.

[3] H. Guo, H. Zhao, H. Niu, Y. Ren, H. Fang, X. Fang, R. Lv, M. Maqbool, S. Bai, *ACS Nano* **2021**, *15*, 6917.

[4] Y. Jia, H. He, Y. Geng, B. Huang, X. Peng, Compos. Sci. Technol. 2017, 145, 55.

[5] L. Tzounis, M. Petousis, S. Grammatikos, N. Vidakis, *Materials (Basel)* **2020**, *13*.

[6] M. Kim, J. H. Jeong, J. Y. Lee, A. Capasso, F. Bonaccorso, S. H. Kang, Y. K. Lee, G. H. Lee, *ACS Appl Mater Interfaces* **2019**, *11*, 11841.

[6] M. Rinaldi, T. Ghidini, F. Nanni, *Polym. Int.* **2021**, *70*, 1080.

[8] M. P. Maldonado, G. M. Pinto, L. C. Costa, G. J. M. Fechine, *J. Appl. Polym. Sci.* **2022**, *139*, 52405.

[9] N. Vidakis, M. Petousis, E. Velidakis, L. Tzounis, N. Mountakis, O. Boura, S. A. Grammatikos, *Adv. Compos. Mater* **2022**, *31*, 630.

[10] G. Spinelli, P. Lamberti, V. Tucci, R. Kotsilkova, E. Ivanov, D. Menseidov, C. Naddeo, V. Romano, L. Guadagno, R. Adami, D. Meisak, D. Bychanok, P. Kuzhir, *Materials (Basel)* **2019**, *12*, 2369.

[11] S. K. Soni, B. Thomas, V. R. Kar, *Materials Today Communications* **2020**, *25*, 101546.
